# CCR5 Targeted Cell Therapy for HIV and Prevention of Viral Escape

**DOI:** 10.3390/v7082816

**Published:** 2015-07-27

**Authors:** Gero Hütter, Josef Bodor, Scott Ledger, Maureen Boyd, Michelle Millington, Marlene Tsie, Geoff Symonds

**Affiliations:** 1 Cellex GmbH, Fiedlerstr. 36, 01307 Dresden, Germany; 2 Department of Cell Therapy, Institute of Hematology and Blood Transfusion, U Nemocnice 1, 128 20 Prague 2, Czech Republic; E-Mail: bodor@uhkt.cz; 3 Faculty of Medicine, University of New South Wales, Sydney 2052 NSW, Australia; E-Mail: s.ledger@amr.org.au; 4 Calimmune, Inc., Los Angeles, CA 90024, USA; E-Mails: maureen.boyd@calimmune.com.au (M.B.); michelle.millington@calimmune.com.au (M.M.); marlene.tsie@calimmuneinc.com (M.T.); geoff.symonds@calimmuneinc.com (G.S.)

**Keywords:** HIV-1, CCR5, CCR5-delta32, tropism, gene therapy, viral escape, chemokine receptor

## Abstract

Allogeneic transplantation with CCR5-delta 32 (CCR5-d32) homozygous stem cells in an HIV infected individual in 2008, led to a sustained virus control and probably eradication of HIV. Since then there has been a high degree of interest to translate this approach to a wider population. There are two cellular ways to do this. The first one is to use a CCR5 negative cell source e.g., hematopoietic stem cells (HSC) to copy the initial finding. However, a recent case of a second allogeneic transplantation with CCR5-d32 homozygous stem cells suffered from viral escape of CXCR4 quasi-species. The second way is to knock down CCR5 expression by gene therapy. Currently, there are five promising techniques, three of which are presently being tested clinically. These techniques include zinc finger nucleases (ZFN), clustered regularly interspaced palindromic repeats/CRISPR-associated protein 9 nuclease (CRISPR/Cas9), transcription activator-like effectors nuclease (TALEN), short hairpin RNA (shRNA), and a ribozyme. While there are multiple gene therapy strategies being tested, in this review we reflect on our current knowledge of inhibition of CCR5 specifically and whether this approach allows for consequent viral escape.

## 1. Introduction

HIV cell entry relies on binding to the CD4 receptor and at least one of two possible chemokine co-receptors: CCR5 and CXCR4. Tropism is the ability of the virus to bind to a specific co-receptor. HIV strains that bind to CCR5 are called R5-tropic and those that bind to CXCR4 are X4-tropic; dual tropic strains are capable of using either CCR5 or CXCR4. Of the two possible co-receptors, CCR5 is the predominant receptor for HIV cell entry. It is important to note that R5-tropic strains are the most commonly transmitted and are predominant during early stages of infection, while X4-tropic strains emerge later with disease progression [1,2,3,4]. A deletion of 32 base pairs within the CCR5 gene leads to a nonfunctional gene product, which is, in turn, not expressed at the cell surface. Individuals with a homozygous CCR5-d32 deletion do not express any CCR5 receptor and are consequently highly protected from infection with HIV-1 without any other obvious harmful effects to their health [5].

In 1996, lack of CCR5 co-receptor at the cell surface was first described as a natural resistance against transmission. Subsequently, several approaches have been undertaken to use this “Achilles heel” of HIV-1 to develop new CCR5 targeted treatment strategies in addition to the established antiretroviral therapy (ART).

While there are many strategies to inhibit HIV infection, here we will focus on methods relating to CCR5 as it is the predominant co-receptor. Methods to down regulate/inhibit synthesis of CCR5 include ZFN, CRISPR/Cas9, TALEN, shRNA, small interfering RNAs (siRNA), antisense RNA, and ribozymes. Methods to prevent surface expression of CCR5 include intrabodies and intrakines [6,7,8,9].

## 2. Strategies to Down Regulate and Inhibit Synthesis of CCR5

1. ZFNs are engineered proteins with zinc finger domains that can bind to targeted regions of DNA and conduct gene editing via double strand DNA breaks. Locations of DNA breakage can undergo either non homologous end joining or homologous recombination by insertion of donor DNA, both of which lead to mutations of the gene [10,11]. Tebas *et al.* 2014 report that they safely used ZFN to modify CCR5 of autologous CD4+ T cells used to treat HIV positive patients [12]. A trial currently recruiting participants uses ZFN to modify CCR5 in CD4+ T cells with increasing doses of cyclophosphamide to promote engraftment (NIH clinical trial NCT01543152).
2. Recently, more advanced techniques in gene suppression have been established. For example TALENs can successfully target sites in the CCR5 loci with less cytotoxicity compared to ZFNs [13]. Similar to ZFNs the TALENs binding domains recognize and cleave specific DNA by using the previously fused endonuclease part of this complex. This can be carried by adenoviruses. Unlike ZFNs, TALENs recognize only one nucleotide instead of three [14]. Mock *et al*. 2015report the use of TALEN to knockout CCR5. This technique was shown to protect CCR5 T cells from R5-tropic HIV [15]. However, it should be noted that Mock *et al.* used transient transfection methods and only report one long term (12 days) exposure to HIV which showed incomplete suppression of HIV replication [15].
3. To combat hostile agents, bacteria harbor an effective defense mechanism: the CRISPR/Cas9 system. It works as an intracellular defense system against plasmids or viral DNA by causing site specific double strand breaks. The CRISPR/Cas9 system was adapted as a molecular tool to break down single human genes. In fact, it has been successfully tested in human cells. There, Kim *et al.* were able to affect 18% of the CCR5 genes, a percentage that may be essential for a successful clinical use [16].
4. siRNAs are small pieces of synthetically derived RNA that guide an endonuclease to cleave a targeted site in mRNA. siRNAs are exogenously synthesized, fragile (21-23-mer short), and prone to quick degradation. They need to be administered in high dosages to reach the targeted RNA of interest. siRNAs have been used to target CCR5 in several studies, however, they resulted in an incomplete inhibition of HIV-1 and off-target effects [17,18]. Even though siRNAs are target specific, viral escape mutants have been documented to make their use less than ideal for clinical applications [19,20].
5. shRNAs differ from siRNAs by virtue of a more stable secondary structure (hairpin loop). Such structure enables researchers to use only a small dose of it to reach the target. Also, shRNAs can be expressed in the target cell’s nucleus via a gene cassette . Lentiviral vectors can efficiently express shRNAs. In fact, they have recently been shown to inhibit HIV in human cells and animal models [21,22,23]. There is currently an open clinical trial that employs a lentiviral vector to express shRNA to CCR5 in combination with C46 (NIH clinical trial NCT01734850).
6. Translation at the mRNA level can be inhibited by antisense RNAs; single stranded complementary RNAs. Li *et al*. 2006 report the downregulation of CCR5 by recombinant adenovirus expressing antisense CCR5 RNA [24]. However, the authors note that the vector is only transiently expressed and, if used for treatment, would require frequent dosing due to elimination by the host’s immune system. Therefore, making this technique less than an ideal gene therapy.
7. Ribozymes are small catalytic RNA molecules that can act like protein enzymes and be engineered to target specific RNA sequences [25,26,27,28]. Three clinical trials have positively showed the safety, feasibility, and long term stability of using ribozymes targeted to tat and tat-vpr HIV elements [27,29,30]. However, the transduction efficiency left room for improvement. Also, none of the trial participants underwent myeloablation. Since then advances have been made in gene transfer technology. Currently, myeloablation is being tested in conjunction with gene therapy to treat HIV. DiGiusto *et al*. 2010 reports a combinatorial approach that includes Tat/Rev shRNA, Tat activation-response region (TAR) decoy, and CCR5 ribozyme used to genetically modify autologous peripheral blood derived CD34+ HSC from AIDS patients [31]. Reports from this ongoing clinical trial revealed that the stability of CCR5 ribozyme was maintained up to 24 months and noted the need for improvement of transduction processes (NIH clinical trial NCT00569985).

## 3. Strategies to Inhibit Cell Surface Expression of CCR5

1. Intrakines are intracellular chemokines capable of targeting newly synthesized CCR5 in the endoplasmic reticulum by blocking transport of CCR5 to the cell surface [7,8,9]. Probably one of the first attempts to inhibit the use of chemokine co-receptors to generate HIV resistant cells was published in 1997. Here, the group used intrakines against CCR5 [32]. However, the major problem in this approach was reported to be an incomplete inhibition of CCR5.
2. In contrast to the use of intrakines, the use of intrabodies provided a more complete inhibition of CCR5. Intrabody is an intracellular single chain variable fragment antibody (scFv) that can bind to a protein of interest potentially rendering it dysfunctional. Steinberger *et al.* 2000 developed a CCR5 specific intrabody capable of blocking surface expression of CCR5, thereby protecting gene modified cells from HIV infection [33].

Allogeneic stem cell transplantation with CCR5-d32/d32 cells in patients with HIV-infection and malignancy has been considered since the late 1990s. When receiving donor cells, it is critical to have properly matched human leukocyte antigens (HLA). Otherwise increasing the risk of rejection by the host’s immune system. The limited availability of HLA-matched unrelated donors has not increased over the last two decades. Only ~1% of Caucasians possess the CCR5 null allele. This makes the approach virtually impossible. To overcome this limitation, StemCyte (Covina, CA, USA), a cord blood bank, in 2001 started to test all of their stored units for CCR5-deletion to offer transplantation centers a CCR5 negative stem cell source. However, after identifying several hundreds of CCR5-d32/d32 units, the probability of finding an appropriately HLA-matched graft with sufficient cell count (>2.5 × 10^7^ total nucleated cells) was still low. Specifically, only approximately 27% of Caucasian patients were appropriately matched [34]. Combined with homozygous CCR5-d32/d32 of 1%, this becomes very infrequent.

The concept of CCR5-depleted HIV cell therapy was given credence by the first successful allogeneic transplantation with a perfect HLA-match from a donor homozygous for the CCR5-d32 deletion. In this patient (the “Berlin patient”) ART was stopped from the time of transplantation and he has stayed free from any viral rebound for a period of seven years. Moreover, even techniques with the highest sensitivity have failed to detect any replication competent viral material, indicating that the patient has received a sterilizing cure of HIV-1 infection [35,36].

A couple of years later, a second patient (the “Essen patient”) received a treatment similar to the “Berlin patient”. In this case, the Essen patient’s viral load was found to rebound during engraftment with an HIV quasi-species which was able to use alternative chemokine receptors (in this case CXCR4) [37]. This incident has raised several questions concerning the feasibility of translating the techniques of CCR5 down-regulation by gene therapy to a larger group of patients. In this review, the problem of viral rebound of non-CCR5 tropic viruses after CCR5 down-regulation is examined and possible strategies to prevent viral escape, the ability of HIV to evade immune responses using multiple mechanisms (e.g., sequestration, latent reservoirs, switch to X4-tropism, epitope mutation or deletion, or exhaustion of cytotoxic T lymphocytes), are discussed.

## 4. Differences between the “Berlin” and the “Essen Patient”

Seven years after the successful treatment of the “Berlin patient”, the results of the “Essen patient” were published in 2014. The feeling of disappointment in both the scientific community and the general public was tangible. These cases were very similar because both patients received HLA matched unrelated stem cells from a CCR5-d32 homozygous donor. However, in the “Essen patient” case, the virus rebounded after transplantation with an X4 strain. Looking into the details of both patients’ cases and the clinical course reveals some important differences (Table 1).

**Table 1:** “Berlin patient” *versus* “Essen patient”. Differences between the “Berlin” and the “Essen” patient receiving a CCR5-delta32 homozygous allogeneic stem cell transplantation.

	Berlin patient	Essen patient
Age, sex	40 years, male	27 years, male
Malignancy	acute myeloid leukemia	anaplastic large T-cell lymphoma
Time between infection and ART	7 years	3 years
Time between infection and Tx	12 years	5 years
Tx regimen	intermediate intensity	myeloablative + 12 Gy TBI
Immunosuppression	ATG, CSA, MTX, MMF	ATG, CSA, MTX,
GVHD	max. grade 1 (skin)	max. grade 1–2 (skin)
Engraftment	day +11	day +39
ART discontinuation	on day of Tx	7 days before Tx
V3 sequence	CIRPNNNTRK**G**IHIGPGR**A**FYTTGEIIGDIRQAHC	CTRPNNNTRK**G**I**PL**GPG**KV**FY**A**T**-**EII**R**DIR**K**A**Y**C
>3 months prior Tx *		
X4 prediction **		
3months prior Tx	capable	intermediate
Immediate prior Tx	nd	capable

* Discrepancy to the HIV type B V3 consensus sequence (CTRPNNNTRKSIHIGPGRFYTTGEIIGDIRQAHC) are marked in bold and underlined. ** Prediction of using the CXCR4 receptor (DNA or RNA according to Geno3Pheno). ART = antiretroviral therapy, ATG = anti-thymocytic globulin, CSA = cyclosporine A, MMF = mycophenolate mofetil, MTX = methotrexate, nd = not done, TBI = total body irradiation, Tx = transplantation.

The clinical course of HIV infection was much more unfavorable in the “Essen patient”. The latency between first diagnosis, initiation of ART, and malignancy was very short. By developing a T-cell lymphoma, the “Essen patient” rapidly developed AIDS. Whereas the “Berlin patient” never had an opportunistic infection and continuously maintained a sufficient CD4 T-cell count before developing leukemia.

Corresponding to the clinical course, in depth analysis of the tropism predicting V3 region of the virus revealed a major alteration in the “Essen patient”. As compared to the consensus sequence, the “Essen patient” had a higher probability to change the tropism from R5 to X4, which was triggered by a few additional mutations.

Another critical difference was that ART was stopped one week before transplantation in the “Essen patient”. It appears that this gave sufficient time for the virus to replicate to an extent that it evolved to use alternative co-receptors. The fact that the “Essen patient” engrafted very late (usually a stable hematopoiesis will be achieved after 2–3 weeks after transplantation) could be evidence for a rapid outgrowth of the mutated virus with consequent cytopathic effects on the expanding hematopoiesis. This could have contributed to the delayed engraftment. Both, uncontrolled viral replication and late engraftment of CCR5 negative cells for nearly six weeks appear to have been contributing factors of tropism change in the “Essen patient” case.

Finally, the “Berlin patient” relapsed from his leukemia one year after the first transplant and received a second transplant from the same donor. It is conceivable that this double-transplantation has enhanced the purging effect of this procedure on HIV reservoirs. However, the “Berlin patient” was HIV free without antiretroviral therapy between these interventions so that eradication might have been achieved before the second transplantation.

The main lesson we have learnt from the “Essen patient” is to continue ART during the conditioning regimen until a stable engraftment and a 100% chimerism is achieved. Concerns that cytotoxic effects of antiretrovirals may cause graft failure are, in our experience, overrated [38]. On the other hand, drug-drug interaction between antiretrovirals and medications during transplantation procedure (e.g., cyclosporine A) are noteworthy and sometimes difficult to handle. Another unsolved problem is that antiretroviral medication is exclusively administered orally which may cause problems in cases where patients develop severe mucositis during their aplasia. Recommendations and guidelines for a safe use of ART during chemotherapy and allogeneic transplantation are necessary to make educated clinical decisions [39].

## 5. HIV Tropism and CCR5 Suppression

CCR5 is the major receptor for HIV cell entry. However, during the time of infection HIV-1 may change its tropism in part (e.g., to CXCR4). This switch is associated with: high viral load, low CD4+ T cell count, AIDS, and ART pre-treatment, and may occur despite suppressed viral load. For CCR5 targeted treatment options, it is essential to understand the preconditions of the tropism shift to avoid viral escape. Our experiences are mainly based on three examples where CCR5 and tropism change were analyzed.

### 5.1. Entry Inhibitors of HIV

In 2007 maraviroc, a competitive CCR5 inhibitor, received clinical approval. Maraviroc is representative of the new HIV drug class (entry inhibitors) that showed additional efficiency in ART pretreated patients [40]. However, viral failure has been observed by re-emergence of X4-tropic viruses with ongoing entry inhibitor use. This phenomenon was intensively studied and most studies found the presence of even very small populations of non-CCR5 using strains of HIV before maraviroc initiation. Evolutionary analysis revealed that X4-tropic viruses do not evolve *de novo* as a result of increased selective pressure, but rather arise from pre-existing populations [41,42]. Therefore, X4-variants of HIV can re-emerge if the R5 viral suppression is incomplete and/or the reservoir size of HIV has not been suppressed to a critical level.

### 5.2. CCR5 Gene Therapy of HIV Disease

The Sangamo trial is the most advanced HIV gene therapy trial in terms of patient recruitment. In this trial, peripheral autologous T-cells were administered after manipulation with a ZFN against CCR5. A group of patients underwent a treatment interruption after cell infusion. All of them rebounded rapidly indicating that the manipulated cells did not exhibit protection from viral replication. The dosage of manipulated CCR5 negative cells was relatively small. Interestingly, one of the patients who was found to be heterozygous for the CCR5-d32 deletion spontaneously developed control of viral replication without any antiretroviral treatment. All candidates for these investigations exclusively harbored R5-tropic strains of HIV prior to enrolment. Unfortunately, there are no data concerning possible shifts in HIV tropism during zinc-finger administration and in the patient who controlled viral replication [12].

### 5.3. Problems Unsolved: Alternative Chemokine Receptors

Generally, people with the natural CCR5-d32/d32 mutation who become infected by HIV are infected by X4-tropic viruses. However, it is well known that under some circumstances HIV can use alternative chemokine receptors. Most interesting in this context is analysis of tropism from HIV-infected CCR5-d32 homozygotes where cases of infection with non-R5-tropic viruses have been described [43,44]. This “back door” of HIV rebound could be a critical limitation for entry-targeted treatment strategies.

## 6. Size of the Reservoir and Probability of Rebound

In patients where antiretroviral medication is discontinued, the rebound generally occurs within a few weeks. Interestingly, relatively rapid rebound is still observed even in patients who have been taking antiretrovirals for a long time (minimization of the reservoir is assumed) [45]. Moreover, in patients with spontaneous suppression of viral replication (elite controllers) there is generally a measurable proviral reservoir. Also, elite controllers may suffer from a spontaneous and unexpected periodic outburst of HIV replication [46].

Most recently, reports of patients with non-detectable viral reservoirs were published and deserve closer attention.

1. Two HIV+ patients received allogeneic stem cell transplantation (CCR5 wild type graft). Both received antiretroviral medication—2.5 and 4.3 years after transplantation. Both developed a continuous sero-deconversion concerning anti-HIV antibodies indicating that no significant replication had occurred in this time. Otherwise the anti-HIV antibodies would have still been measurable. Furthermore, tissue samples and outgrowth assays from peripheral blood were all negative. It has been speculated that allogeneic transplantation led to elimination of the viral reservoir by turnover of latently infected cells in combination with (a postulated) cytotoxic effect from graft T-cells against the reservoirs (graft *versus* HIV effect). However, both patients rebounded with HIV after three and seven weeks, respectively. Interestingly, the time between discontinuation of medication and rebound was unusually long, indicating that both patients had a very small reservoir but had not eradicated the virus and that latently infected cells “hid” in undetectable niches [47,48].
2. A perinatal infected child received antiviral therapy rapidly (30 h) after delivery and was taken off medication after 18 months. Surprisingly, the child was found without HIV replication after ART discontinuation. Occasionally very small traces of virus material were detected but no replication competent virus was found. The patient displayed no immunological reaction in terms of anti-HIV antibody production. It was assumed that early initiation of ART led to a minimized reservoir and that the immune system was able to control this small number of infected cells. However, approximately 27.6 months after discontinuation of ART the child was found with active HIV replication [49].

A functional cure was assumed in all three patients by minimizing the size of the latent reservoir and thereby achieving viral control. Unfortunately, all three patients rebounded with HIV after unusually long periods of time indicating that viral reemergence can occur from very small cell sources which might be far beyond our current abilities of detection. Assays for quantifying the latent reservoir continue to be developed and optimized. Outgrowth assays using ELISA take 14 days while newer RT-PCR assays take only seven days and are more sensitive [50]. Despite advancements in quantifying the reservoir, their accuracy is uncertain and the source of latently infected cells remains unknown.

## 7. Strategies to Minimize the Viral Reservoir

As reported above, the size of the viral reservoir could contribute to the probability and the time point of viral replication after ART discontinuation. Therefore, strategies to minimize the reservoir will be critical, such as enhancing the potency of CCR5 driven therapies.

### 7.1. Chemotherapy

Chemotherapy alone or in combination with irradiation is used routinely during cancer treatment as well as autologous and allogeneic stem cell transplantation. The myelosuppressive effect of chemotherapy is sustained but usually only temporarily. Some chemotherapeutics (e.g., fludarabine) cause more cell line-specific and lasting effects. Experiences with HIV+ patients receiving high dose chemotherapy and autologous stem cell transplantation have revealed a lack of sustained effect on the size of the viral reservoir [51].

### 7.2. Using HIV Cytopathic Effects

HIV has a cytopathic effect on cells, which means that replication and externalization of HIV particles may lead to a consequent cell death. In uncontrolled infection, reduction of the pool of infected cells is compensated by newly infected target cells. Introduction of ART provided some hope in that latently infected cells had a significant turnover and that continuation of ART would lead to stepwise eradication of HIV. Strategies to enhance the turnover rate were proposed when it became obvious that the half-life of the cell turnover is much longer than expected (due to resting and non-replicating cell sources) and that eradication could not be achieved during a normal life-span. Under the terms “shock and kill” or “kick and kill” different medications were tested. These medications were able to “kick/shock” the latently infected cells to replicate their virus followed by the elimination (“kill”) of the replicating cell. Concurrently, antiretrovirals, including entry inhibitors, were administered to prevent reinfection of cells. For this approach, histone deacetylase inhibitors, inhibitors of extra-terminal proteins, and protein kinase C activators have been tested [52]. However, first clinical experiences with vorinostat, a histone deacetylase inhibitor exhibited evidence that the virus can be “kicked” out of the reservoir but failed to show any effect on the size of the viral reservoir [53].

### 7.3. Agents Toxic to Viral Reservoir

Several agents have been shown to have cytotoxic effects on latently infected cells. One promising candidate is auranofin, a gold complex initially developed for treatment of rheumatic arthritis which is through to inhibit reduction/oxidation (redox) enzymes who are essential to many cellular processes, particularly in maintaining the intracellular levels of reactive oxygen species (ROS). Controlling the level of ROS to prevent the resulting DNA damage is critical for the survival of all cell types, including cancer cells, parasites and memory T cells that harbour proviral HIV DNA. Auranofin has pro-apoptotic effects through a proposed caspase pathway and down-modulation of CD27 in non-activated central and transitional memory T-cells (T_CM_ and T_TM_, respectively). CD27 is a marker of memory T cells with long-lived phenotypes harboring provirus [54].

An early clinical trial with auranofin in patients infected with HIV was announced from the Vaccine and Gene Therapy Institute, Florida, USA but was withdrawn prior to enrollment (NIH clinical trial NCT02176135).

### 7.4. Gene Therapy

In addition to gene therapy being feasible in targeting HIV entry mechanism, it may be useful in targeting the latently infected cells to reduce the size of the reservoir. In 2007 a new approach of purging infected cells attracted attention. This approach used a highly specific tre-recombinase. This enzyme was derived from another enzyme with the ability to cut DNA regions with a similar sequence as the long terminal repeats (LTR) found in HIV-1 insertion sites. By insertion of the tre-recombinase into the infected cell, the provirus can be completely removed [55].

Most recently, another approach using Cas9/guide RNA (gRNA) has been published. Here, only a fragment of the integrated provirus was successfully tested. By interfering with the HIV-1 LTR U3 region, Cas9/gRNA transfection inactivated HIV gene expression and replication of latently infected cells effectively [56].

## 8. Strategies to Overcome Viral Rebound

### 8.1. Additional HIV Entry Inhibition

In light of mixed results attendant CCR5 down-regulation (as previously described), a case can be made for dual entry inhibition. One possibility is to use an agent that has an additional effect on CCR5 down-regulation. The HIV-fusion inhibitor C46 (M87o), developed by the von Laer laboratory, has shown strong anti-HIV activity in tissue culture systems and in non-human primates [57,58,59]. C46, comprised of 46 amino acids, is derived from the second heptad repeat of the HIV-1 envelope glycoprotein gp41, and effectively inhibits fusion of the viral and cellular membranes during virus entry. The 36 C-terminal amino acids of C46 correspond to the fusion-inhibitory peptide C36 (T-20/enfurvitide), the first HIV-fusion inhibitor approved for clinical use. C46 is expressed as a fusion protein with an N-terminal signal peptide, which targets the peptide through the endoplasmic reticulum to the cell surface and a C-terminal linker followed by a membrane-spanning domain. Safety of this inhibitor has been shown *in vitro* and in a clinical trial [60]. A lentiviral vector comprised of the C46 fusion inhibitor and a shRNA to CCR5 (LVsh5/C46) has been shown to have a synergistic effect in inhibition of HIV replication in T cell lines, peripheral blood mononuclear cells as well as in a humanized mouse model *in vivo* [21]. Experiments conducted with LVsh5/C46 were used to transduce Molt 4/CCR5 cells and then challenged with HIV Bal (R5-tropic). Results from triplicate experiments showed increasing gene marking over time (10 to >75%) and no evidence of escape mutants as shown by PCR analysis on HR1, HR2, and V3 loop regions for up to nine weeks [61].

Another combination therapy currently in clinical trial uses a triple combination of shRNA targeting tat and rev mRNAs of HIV-1, anti-CCR5 ribozyme, and a nucleolar-localizing transactivation response (TAR) decoy (NIH clinical trial NCT00569985) [31,62,63]. Tat and rev are regulatory proteins necessary for HIV to replicate, but shRNAs targeted to them have shown anti-HIV-1 activity in human cells and a humanized mouse model [62,63]. Tat must bind to TAR for transcription. As such, use of the TAR decoy, which can mimic tat binding to TAR, was shown to neutralize its activity [64]. Together with knock down of CCR5, via a ribozyme, this triple combination therapy targets both HIV and cellular elements through different mechanisms. Multiple combination therapy increases the ability to inhibit HIV on several levels and is hoped to suppress any chances for viral escape.

### 8.2. CXCR4 Blockage

Independent from the development of maraviroc as a CCR5 inhibitor, plerixafor a CXCR4 inhibitor was first synthesized in the late 1980s. Plerixafor later became an attractive potential HIV drug. Even though the effect on HIV was quite disappointing, its ability to block the hematopoietic stem cell homing mechanism was noteworthy. Today, plerixafor is administered as a safe and efficient stem cell mobilizer during autologous or allogeneic stem cell collection [65].

**Figure 1:**
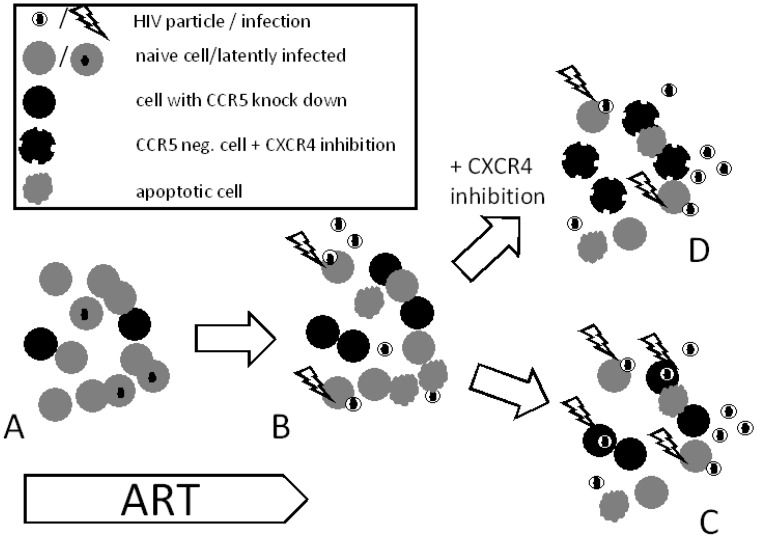
Selective advantage of dual entry inhibition. (**A)** Autologous cells with CCR5 down regulation will be reinfused into a patient ongoing ART; (**B)** ART is discontinued allowing HIV to replicate and infect naïve cells (CCR5+ cells). In theory, based on the cytopathic effect of HIV, CCR5 negative cells will become enriched; (**C)** apoptosis of infected cells decreases CCR5 as a potential target for cell entry. By increasing the selective pressure, HIV may switch tropism and enter CCR5 negative cells by using alternative chemokine receptors like CXCR4; (**D)** Dual entry inhibition (CCR5 negative and CXCR4 inhibited cells) could prevent HIV from entering the cells and thereby infection.

The use of plerixafor in combination with gene therapy has not yet been tested. Principally, the use of plerixafor or other CXCR4 inhibitors might be useful in enrichment of CCR5 manipulated cells to avoid outgrowth of alternative quasi-species of HIV (Figure 1). The current concept of gene therapy proposes that only a relatively limited proportion of cells can be transduced and, thereby, exhibit resistance. In this scenario, other “unprotected” cells would have a disadvantage and be reduced in numbers by the cytopathic effect of HIV. As a consequence, the cells with the protective agent would become enriched and may go on to form a major cell population. In this case, viral replication would act as a selective agent allowing protected population of cells to expand before any untoward effects of HIV replication manifest themselves.

Newly developed CXCR4 inhibitors such as burixafor (TG-0054) provide alternatives to plerixafor. Burixafor exhibits higher efficiency and favorable toxicity as compared to Plerixafor. Burixafor may also be able to contribute to a CCR5 targeted treatment approach [66].

Recently, in an *in vitro* experiment ZFNs were used to disrupt both CCR5 and CXCR4. [32]. In a cell line model only the double manipulated group showed resistance against R5 and X4 strains, indicating a highly selective advantage against the cytopathic effect of HIV infection. However, knock-down of CXCR4 *in vivo* is risky because this receptor appears to be essential in immune responses and also serves as a vital factor for humans. The practicality of this approach needs further exploration [67].

### 8.3. Chemokine Receptor Down-regulation

Density of chemokine receptors on the cell surface may contribute to susceptibility of cells to HIV. In turn, reverse down-regulation can also serve as a protective factor [68,69]. It is known that expression of chemokine receptors is subject to tight regulation. One example of such regulation is prostaglandin E_2_ (PGE_2_)-mediated transcriptional attenuation of CCR5. It has been demonstrated that inducible regulatory T cells (iTregs) express cyclooxygenase-2 (COX-2) and produce copious amounts of PGE_2_ upon differentiation in the periphery [70]. In contrast to naturally occurring CD4^+^CD25^+^Foxp3^+^ Tregs (nTregs), which develop in the thymus and do not express COX-2, iTregs develop in peripheral lymphoid tissues. iTregs’ COX-2-mediated PGE_2_ production can substantially influence epigenetic down-regulation of CCR5 surface expression via bystander effect both on CD4^+^ T cells as well as myeloid cells. Importantly, it has been shown that PGE_2_ and its more stable derivative dimethyl PGE_2_ (dmPGE_2_) are critical regulators of HSC homeostasis [71]. Recent data from clinical trials has shown that brief *ex vivo* modulation with dmPGE_2_ could improve patient outcomes by increasing the “effective dose” of HSCs in allogeneic transplantation, thus opening the possibility that the same treatment could also epigenetically down-regulate CCR5 expression. Similar effects have also been proposed for PGE_2_-mediated down-regulation of CXCR-4.

## 9. Summary and Outlook

Today, HIV treatment has entered a new era. In light of the success of ART in controlling infection and extending the life-span of HIV-infected people, lack of an efficient vaccine is the major obstacle to HIV prevention; perhaps now is the time to consider eradicating HIV. The case of the “Berlin patient” opened a window to the possibility of cell-based therapy. However, there are some limitations in the field of cell-based gene therapy. Gene therapy methods are currently being tested clinically.

New reports of cases where viral control or eradication of HIV might be achieved are prominent today. These reports, however, may diverge the discussions on HIV cure leading to false expectations and hasty conclusions. After all, every new development is still just a piece of the puzzle instead of a definitive solution to HIV treatment optimization.

To our current knowledge, protection from HIV re-emergence after CCR5 targeted therapy can be achieved by suppressing the chemokine receptor to low levels, thus mimicking the nearly perfect protection against transmission in CCR5-d32 homozygous individuals. Inhibition of viral replication during this phase, as well as CCR5-independent entry inhibitors, may prevent rebound of HIV quasi-species using alternative chemokine receptors. Other strategies, such as reducing the size of the HIV reservoir, may facilitate this effect but as of now are entirely hypothetical.

## References

[1] Doms R.W. (2000). Beyond receptor expression: The influence of receptor conformation, density, and affinity in HIV-1 infection. Virology.

[2] Weiss R.A. (2013). Thirty years on: HIV receptor gymnastics and the prevention of infection. BMC Biol..

[3] Connor R.I., Sheridan K.E., Ceradini D., Choe S., Landau N.R. (1997). Change in coreceptor use correlates with disease progression in HIV-1-infected individuals. J. Exp. Med..

[4] Scarlatti G., Tresoldi E., Bjorndal A., Fredriksson R., Colognesi C., Deng H.K., Malnati M.S., Plebani A., Siccardi A.G., Littman D.R. (1997). *In vivo* evolution of HIV-1 co-receptor usage and sensitivity to chemokine-mediated suppression. Nat. Med..

[5] Samson M., Libert F., Doranz B.J., Rucker J., Liesnard C., Farber C.M., Saragosti S., Lapoumeroulie C., Cognaux J., Forceille C. (1996). Resistance to HIV-1 infection in caucasian individuals bearing mutant alleles of the CCR-5 chemokine receptor gene. Nature.

[6] Nazari R., Joshi S. (2008). CCR5 as target for HIV-1 gene therapy. Curr. Gene Ther..

[7] Schroers R., Davis C.M., Wagner H.J., Chen S.Y. (2002). Lentiviral transduction of human T-lymphocytes with a RANTES intrakine inhibits human immunodeficiency virus type 1 infection. Gene Ther..

[8] Bai X., Chen J.D., Yang A.G., Torti F., Chen S.Y. (1998). Genetic co-inactivation of macrophage- and T-tropic HIV-1 chemokine coreceptors CCR-5 and CXCR-4 by intrakines. Gene Ther..

[9] Luis Abad J., Gonzalez M.A., del Real G., Mira E., Manes S., Serrano F., Bernad A. (2003). Novel interfering bifunctional molecules against the CCR5 coreceptor are efficient inhibitors of HIV-1 infection. Mol. Ther..

[10] Li L., Wu L.P., Chandrasegaran S. (1992). Functional domains in Fok I restriction endonuclease. Proc. Natl. Acad. Sci. USA.

[11] Kim Y.G., Cha J., Chandrasegaran S. (1996). Hybrid restriction enzymes: Zinc finger fusions to Fok I cleavage domain. Proc. Natl. Acad. Sci. USA.

[12] Tebas P., Stein D., Tang W.W., Frank I., Wang S.Q., Lee G., Spratt S.K., Surosky R.T., Giedlin M.A., Nichol G. (2014). Gene editing of CCR5 in autologous CD4 T cells of persons infected with HIV. N. Engl. J. Med..

[13] Mussolino C., Morbitzer R., Lutge F., Dannemann N., Lahaye T., Cathomen T. (2011). A novel TALE nuclease scaffold enables high genome editing activity in combination with low toxicity. Nucleic Acids Res..

[14] Holkers M., Maggio I., Liu J., Janssen J.M., Miselli F., Mussolino C., Recchia A., Cathomen T., Goncalves M.A. (2013). Differential integrity of TALE nuclease genes following adenoviral and lentiviral vector gene transfer into human cells. Nucleic Acids Res..

[15] Mock U., Machowicz R., Hauber I., Horn S., Abramowski P., Berdien B., Hauber J., Fehse B. (2015). mRNA transfection of a novel TAL effector nuclease (TALEN) facilitates efficient knockout of HIV co-receptor CCR5. Nucleic Acids Res..

[16] Cho S.W., Kim S., Kim J.M., Kim J.S. (2013). Targeted genome engineering in human cells with the Cas9 RNA-guided endonuclease. Nat. Biotechnol..

[17] Martinez M.A., Gutierrez A., Armand-Ugon M., Blanco J., Parera M., Gomez J., Clotet B., Este J.A. (2002). Suppression of chemokine receptor expression by RNA interference allows for inhibition of HIV-1 replication. AIDS.

[18] Qin X.F., An D.S., Chen I.S., Baltimore D. (2003). Inhibiting HIV-1 infection in human T cells by lentiviral-mediated delivery of small interfering RNA against CCR5. Proc. Natl. Acad. Sci. USA.

[19] Boden D., Pusch O., Lee F., Tucker L., Ramratnam B. (2003). Human immunodeficiency virus type 1 escape from RNA interference. J. Virol..

[20] Das A.T., Brummelkamp T.R., Westerhout E.M., Vink M., Madiredjo M., Bernards R., Berkhout B. (2004). Human immunodeficiency virus type 1 escapes from RNA interference-mediated inhibition. J. Virol..

[21] Burke B.P., Levin B.R., Zhang J., Sahakyan A., Boyer J., Carroll M.V., Colon J.C., Keech N., Rezek V., Bristol G. (2015). Engineering Cellular Resistance to HIV-1 Infection *In Vivo* Using a Dual Therapeutic Lentiviral Vector. Mol. Ther. Nucleic Acids.

[22] Wolstein O., Boyd M., Millington M., Impey H., Boyer J., Howe A., Delebecque F., Cornetta K., Rothe M., Baum C. (2014). Preclinical safety and efficacy of an anti-HIV-1 lentiviral vector containing a short hairpin RNA to CCR5 and the C46 fusion inhibitor. Mol. Ther. Methods Clin. Dev..

[23] Shimizu S., Kamata M., Kittipongdaja P., Chen K.N., Kim S., Pang S., Boyer J., Qin F.X., An D.S., Chen I.S. (2009). Characterization of a potent non-cytotoxic shRNA directed to the HIV-1 co-receptor CCR5. Genet. Vaccines Ther..

[24] Li W., Yu M., Bai L., Bu D., Xu X. (2006). Downregulation of CCR5 expression on cells by recombinant adenovirus containing antisense CCR5, a possible measure to prevent HIV-1 from entering target cells. J. Acquir. Immune Defic. Syndr..

[25] Sarver N., Cantin E.M., Chang P.S., Zaia J.A., Ladne P.A., Stephens D.A., Rossi J.J. (1990). Ribozymes as potential anti-HIV-1 therapeutic agents. Science.

[26] Rossi J.J. (2007). Targeted cleavage: Tuneable cis-cleaving ribozymes. Proc. Natl. Acad. Sci. USA.

[27] Macpherson J.L., Boyd M.P., Arndt A.J., Todd A.V., Fanning G.C., Ely J.A., Elliott F., Knop A., Raponi M., Murray J. (2005). Long-term survival and concomitant gene expression of ribozyme-transduced CD4+ T-lymphocytes in HIV-infected patients. J. Gene Med..

[28] Sun L.Q., Wang L., Gerlach W.L., Symonds G. (1995). Target sequence-specific inhibition of HIV-1 replication by ribozymes directed to tat RNA. Nucleic Acids Res..

[29] Amado R.G., Mitsuyasu R.T., Rosenblatt J.D., Ngok F.K., Bakker A., Cole S., Chorn N., Lin L.S., Bristol G., Boyd M.P. (2004). Anti-human immunodeficiency virus hematopoietic progenitor cell-delivered ribozyme in a phase I study: Myeloid and lymphoid reconstitution in human immunodeficiency virus type-1-infected patients. Hum. Gene Ther..

[30] Mitsuyasu R.T., Merigan T.C., Carr A., Zack J.A., Winters M.A., Workman C., Bloch M., Lalezari J., Becker S., Thornton L. (2009). Phase 2 gene therapy trial of an anti-HIV ribozyme in autologous CD34+ cells. Nat. Med..

[31] DiGiusto D.L., Krishnan A., Li L., Li H., Li S., Rao A., Mi S., Yam P., Stinson S., Kalos M. (2010). RNA-based gene therapy for HIV with lentiviral vector-modified CD34(+) cells in patients undergoing transplantation for AIDS-related lymphoma. Sci. Transl. Med..

[32] Yang A.G., Bai X., Huang X.F., Yao C., Chen S. (1997). Phenotypic knockout of HIV type 1 chemokine coreceptor CCR-5 by intrakines as potential therapeutic approach for HIV-1 infection. Proc. Natl. Acad. Sci. USA.

[33] Steinberger P., Andris-Widhopf J., Buhler B., Torbett B.E., Barbas C.F. (2000). Functional deletion of the CCR5 receptor by intracellular immunization produces cells that are refractory to CCR5-dependent HIV-1 infection and cell fusion. Proc. Natl. Acad. Sci. USA.

[34] Petz L.D., Redei I., Bryson Y., Regan D., Kurtzberg J., Shpall E., Gutman J., Querol S., Clark P., Tonai R. (2013). Hematopoietic cell transplantation with cord blood for cure of HIV infections. Biol. Blood Marrow Transplant..

[35] Hütter G., Nowak D., Mossner M., Ganepola S., Mussig A., Allers K., Schneider T., Hofmann J., Kucherer C., Blau O. (2009). Long-term control of HIV by CCR5 Delta32/Delta32 stem-cell transplantation. N. Engl. J. Med..

[36] Yukl S.A., Boritz E., Busch M., Bentsen C., Chun T.W., Douek D., Eisele E., Haase A., Ho Y.C., Hutter G. (2013). Challenges in detecting HIV persistence during potentially curative interventions: A study of the Berlin patient. PLoS Pathog..

[37] Kordelas L., Verheyen J., Beelen D.W., Horn P.A., Heinold A., Kaiser R., Trenschel R., Schadendorf D., Dittmer U., Esser S. (2014). Shift of HIV tropism in stem-cell transplantation with CCR5 Delta32 mutation. N. Engl. J. Med..

[38] Hutter G., Zaia J.A. (2011). Allogeneic haematopoietic stem cell transplantation in patients with human immunodeficiency virus: The experiences of more than 25 years. Clin. Exp. Immunol..

[39] Flepisi B.T., Bouic P., Sissolak G., Rosenkranz B. (2014). Drug-drug interactions in HIV positive cancer patients. Biomed. Pharmacother..

[40] Fatkenheuer G., Nelson M., Lazzarin A., Konourina I., Hoepelman A.I., Lampiris H., Hirschel B., Tebas P., Raffi F., Trottier B. (2008). Subgroup analyses of maraviroc in previously treated R5 HIV-1 infection. N. Engl. J. Med..

[41] Westby M., Lewis M., Whitcomb J., Youle M., Pozniak A.L., James I.T., Jenkins T.M., Perros M., van der Ryst E. (2006). Emergence of CXCR4-using human immunodeficiency virus type 1 (HIV-1) variants in a minority of HIV-1-infected patients following treatment with the CCR5 antagonist maraviroc is from a pretreatment CXCR4-using virus reservoir. J. Virol..

[42] Archer J., Braverman M.S., Taillon B.E., Desany B., James I., Harrigan P.R., Lewis M., Robertson D.L. (2009). Detection of low-frequency pretherapy chemokine (CXC motif) receptor 4 (CXCR4)-using HIV-1 with ultra-deep pyrosequencing. AIDS.

[43] Gray L., Churchill M.J., Keane N., Sterjovski J., Ellett A.M., Purcell D.F., Poumbourios P., Kol C., Wang B., Saksena N.K. (2006). Genetic and functional analysis of R5X4 human immunodeficiency virus type 1 envelope glycoproteins derived from two individuals homozygous for the CCR5delta32 allele. J. Virol..

[44] Henrich T.J., Hanhauser E., Hu Z., Stellbrink H.J., Noah C., Martin J.N., Deeks S.G., Kuritzkes D.R., Pereyra F. (2015). Viremic control and viral coreceptor usage in two HIV-1-infected persons homozygous for CCR5 Delta32. AIDS.

[45] Davey R.T., Bhat N., Yoder C., Chun T.W., Metcalf J.A., Dewar R., Natarajan V., Lempicki R.A., Adelsberger J.W., Miller K.D. (1999). HIV-1 and T cell dynamics after interruption of highly active antiretroviral therapy (HAART) in patients with a history of sustained viral suppression. Proc. Natl. Acad. Sci. USA.

[46] Cortes F.H., Passaes C.P., Bello G., Teixeira S.L., Vorsatz C., Babic D., Sharkey M., Grinsztejn B., Veloso V., Stevenson M. (2015). HIV controllers with different viral load cutoff levels have distinct virologic and immunologic profiles. J. Acquir. Immune Defic. Syndr..

[47] Henrich T.J., Hu Z., Li J.Z., Sciaranghella G., Busch M.P., Keating S.M., Gallien S., Lin N.H., Giguel F.F., Lavoie L. (2013). Long-term reduction in peripheral blood HIV type 1 reservoirs following reduced-intensity conditioning allogeneic stem cell transplantation. J. Infect. Dis..

[48] Henrich T.J., Hanhauser E., Marty F.M., Sirignano M.N., Keating S., Lee T.H., Robles Y.P., Davis B.T., Li J.Z., Heisey A. (2014). Antiretroviral-free HIV-1 remission and viral rebound after allogeneic stem cell transplantation: Report of 2 cases. Ann. Intern. Med..

[49] Luzuriaga K., Gay H., Ziemniak C., Sanborn K.B., Somasundaran M., Rainwater-Lovett K., Mellors J.W., Rosenbloom D., Persaud D. (2015). Viremic relapse after HIV-1 remission in a perinatally infected child. N. Engl. J. Med..

[50] Laird G.M., Eisele E.E., Rabi S.A., Lai J., Chioma S., Blankson J.N., Siliciano J.D., Siliciano R.F. (2013). Rapid quantification of the latent reservoir for HIV-1 using a viral outgrowth assay. PLoS Pathog..

[51] Zaia J.A., Forman S.J. (2013). Transplantation in HIV-infected subjects: Is cure possible?. Hematology Am. Soc. Hematol. Educ. Program.

[52] Archin N.M., Margolis D.M. (2014). Emerging strategies to deplete the HIV reservoir. Curr. Opin. Infect. Dis..

[53] Elliott J.H., Wightman F., Solomon A., Ghneim K., Ahlers J., Cameron M.J., Smith M.Z., Spelman T., McMahon J., Velayudham P. (2014). Activation of HIV transcription with short-course vorinostat in HIV-infected patients on suppressive antiretroviral therapy. PLoS Pathog..

[54] Chirullo B., Sgarbanti R., Limongi D., Shytaj I.L., Alvarez D., Das B., Boe A., DaFonseca S., Chomont N., Liotta L. (2013). A candidate anti-HIV reservoir compound, auranofin, exerts a selective 'anti-memory' effect by exploiting the baseline oxidative status of lymphocytes. Cell Death Dis..

[55] Sarkar I., Hauber I., Hauber J., Buchholz F. (2007). HIV-1 proviral DNA excision using an evolved recombinase. Science.

[56] Hu W., Kaminski R., Yang F., Zhang Y., Cosentino L., Li F., Luo B., Alvarez-Carbonell D., Garcia-Mesa Y., Karn J. (2014). RNA-directed gene editing specifically eradicates latent and prevents new HIV-1 infection. Proc. Natl. Acad. Sci. USA.

[57] Hildinger M., Dittmar M.T., Schult-Dietrich P., Fehse B., Schnierle B.S., Thaler S., Stiegler G., Welker R., von Laer D. (2001). Membrane-anchored peptide inhibits human immunodeficiency virus entry. J. Virol..

[58] Schambach A., Schiedlmeier B., Kuhlcke K., Verstegen M., Margison G.P., Li Z., Kamino K., Bohne J., Alexandrov A., Hermann F.G. (2006). Towards hematopoietic stem cell-mediated protection against infection with human immunodeficiency virus. Gene Ther..

[59] Younan P.M., Polacino P., Kowalski J.P., Peterson C.W., Maurice N.J., Williams N.P., Ho O., Trobridge G.D., von Laer D., Prlic M. (2013). Positive selection of mC46-expressing CD4+ T cells and maintenance of virus specific immunity in a primate AIDS model. Blood.

[60] Van Lunzen J., Glaunsinger T., Stahmer I., von Baehr V., Baum C., Schilz A., Kuehlcke K., Naundorf S., Martinius H., Hermann F. (2007). Transfer of autologous gene-modified T cells in HIV-infected patients with advanced immunodeficiency and drug-resistant virus. Mol. Ther..

[61] Ledger S., Howe A., Ong A., Boyd M., Millington M., Savkovic B., Murray J., Symonds G. (2015). Entry inhibitor gene modifieid T cells show selective survival advantage against HIV infection. Hum. Gene Ther..

[62] Li M.J., Kim J., Li S., Zaia J., Yee J.K., Anderson J., Akkina R., Rossi J.J. (2005). Long-term inhibition of HIV-1 infection in primary hematopoietic cells by lentiviral vector delivery of a triple combination of anti-HIV shRNA, anti-CCR5 ribozyme, and a nucleolar-localizing TAR decoy. Mol. Ther..

[63] Anderson J., Li M.J., Palmer B., Remling L., Li S., Yam P., Yee J.K., Rossi J., Zaia J., Akkina R. (2007). Safety and efficacy of a lentiviral vector containing three anti-HIV genes—CCR5 ribozyme, tat-rev siRNA, and TAR decoy—in SCID-hu mouse-derived T cells. Mol. Ther..

[64] Michienzi A., Li S., Zaia J.A., Rossi J.J. (2002). A nucleolar TAR decoy inhibitor of HIV-1 replication. Proc. Natl. Acad. Sci. USA.

[65] Herbert K.E., Demosthenous L., Wiesner G., Link E., Westerman D.A., Came N., Ritchie D.S., Harrison S., Seymour J.F., Prince H.M. (2014). Plerixafor plus pegfilgrastim is a safe, effective mobilization regimen for poor or adequate mobilizers of hematopoietic stem and progenitor cells: A phase I clinical trial. Bone Marrow Transplant..

[66] Hsu W.T., Jui H.Y., Huang Y.H., Su M.Y., Wu Y.W., Tseng W.Y., Hsu M.C., Chiang B.L., Wu K.K., Lee C.M. (2015). CXCR4 Antagonist TG-0054 Mobilizes Mesenchymal Stem Cells, Attenuates Inflammation, and Preserves Cardiac Systolic Function in a Porcine Model of Myocardial Infarction. Cell Transplant..

[67] Didigu C.A., Wilen C.B., Wang J., Duong J., Secreto A.J., Danet-Desnoyers G.A., Riley J.L., Gregory P.D., June C.H., Holmes M.C. (2014). Simultaneous zinc-finger nuclease editing of the HIV coreceptors ccr5 and cxcr4 protects CD4+ T cells from HIV-1 infection. Blood.

[68] Reynes J., Portales P., Segondy M., Baillat V., Andre P., Avinens O., Picot M.C., Clot J., Eliaou J.F., Corbeau P. (2001). CD4 T cell surface CCR5 density as a host factor in HIV-1 disease progression. AIDS.

[69] Lin Y.L., Mettling C., Portales P., Reynes J., Clot J., Corbeau P. (2002). Cell surface CCR5 density determines the postentry efficiency of R5 HIV-1 infection. Proc. Natl. Acad. Sci. USA.

[70] Mahic M., Yaqub S., Johansson C.C., Tasken K., Aandahl E.M. (2006). FOXP3+CD4+CD25+ adaptive regulatory T cells express cyclooxygenase-2 and suppress effector T cells by a prostaglandin E2-dependent mechanism. J. Immunol..

[71] Cutler C., Multani P., Robbins D., Kim H.T., Le T., Hoggatt J., Pelus L.M., Desponts C., Chen Y.B., Rezner B. (2013). Prostaglandin-modulated umbilical cord blood hematopoietic stem cell transplantation. Blood.

